# Contrasting functional responses of resident Kupffer cells and recruited liver macrophages to irradiation and liver X receptor stimulation

**DOI:** 10.1371/journal.pone.0254886

**Published:** 2021-07-23

**Authors:** Takuya Ishikiriyama, Hiroyuki Nakashima, Kaori Endo-Umeda, Masahiro Nakashima, Seigo Ito, Manabu Kinoshita, Masami Ikarashi, Makoto Makishima, Shuhji Seki

**Affiliations:** 1 Department of Immunology and Microbiology, National Defense Medical College, Tokorozawa, Saitama, Japan; 2 Division of Biochemistry, Department of Biomedical Sciences, Nihon University School of Medicine, Itabashi, Tokyo, Japan; 3 Department of Nephrology and Endocrinology, National Defense Medical College, Tokorozawa, Saitama, Japan; Universite du Quebec a Montreal, CANADA

## Abstract

In the murine liver, there are two major macrophage populations, namely resident Kupffer cells (resKCs) with phagocytic activity and recruited macrophages (recMφs) with cytokine-producing capacity. This study was performed to clarify the functional differences between these two populations, focusing on their susceptibility to radiation and response to stimulation via liver X receptors (LXRs), which are implicated in cholesterol metabolism and immune regulation. Liver mononuclear cells (MNCs) were obtained from C57BL/6 (WT) mice with or without 2 Gy irradiation, and the phagocytic activity against *Escherichia coli (E*. *coli)* as well as TNF-α production were compared between the two macrophage populations. To assess LXR functions, phagocytosis, TNF-α production, and endocytosis of acetylated low-density lipoprotein (LDL) were compared after synthetic LXR ligand stimulation. Furthermore, LXRα/β knockout (KO) mice and LXRα KO mice were compared with WT mice. Irradiation decreased intracellular TNF-α production by recMφs but did not affect the phagocytic activity of resKCs. *In vitro* LXR stimulation enhanced *E*. *coli* phagocytosis by resKCs but decreased *E*. *coli*-stimulated TNF-α production by recMφs. Phagocytic activity and acetylated LDL endocytosis were decreased in both LXRα/β KO mice and LXRα KO mice, with serum TNF-α levels after *E*. *coli* injection in the former being higher than those in WT mice. In conclusion, resKCs and recMφs exhibited different functional features in response to radiation and LXR stimulation, highlighting their distinct roles liver immunity and lipid metabolism.

## Introduction

Various innate immune cell types can be found in the liver, including natural killer (NK) cells, natural killer T (NKT) cells, and macrophages. Among these, the liver resident macrophages, Kupffer cells, are abundant and contribute to systemic defense, removing most invading bacteria in the circulation [1]. We previously reported the existence of two distinct types of Kupffer cells/macrophages in the liver [2, 3]. One is F4/80+CD68+ resident Kupffer cells (resKCs), which have been previously reported to differentiate from the fetal yolk sac [2, 4–6]. The other is F4/80+CD11b+ recruited Kupffer cells/macrophages (recMφs), which develop from bone marrow monocytes [2, 3]. ResKCs phagocytose and kill bacteria through reactive oxygen species (ROS) production within the phagolysosome. Following phagolysis, they produce chemokines, such as MCP-1, attracting more recMφs into the liver. RecMφs also produce cytokines (TNF-α and IL-12), triggering inflammatory responses in the liver [2]. The co-operation of these two cell populations is important for effective bacterial removal. One of the features characteristic to both is their sensitivity to radiation. In a previous study, we reported that resKCs are radio-resistant, while recMφs are radiosensitive [2]. Repeated exposure to low-dose radiation suppressed recMφ recruitment and ameliorated chronic inflammation in the liver [7]. In this study, we sought to confirm the effect of low-dose irradiation on anti-bacterial immunity. In clinical practice, systemic radiation is usually administered at low doses in order to prevent adverse effects. Thus, confirming the effect of low-dose irradiation on each cell population is also important from a clinical perspective.

TNF-α produced by recMφs is crucial for host defense against infection but also plays a pivotal role in the induction of inflammation and tissue/organ damage associated with septic shock [8]. TNF-α production in the liver is crucial in the pathogenesis of diabetes and non-alcoholic steatohepatitis (NASH) [9, 10]. We previously reported that a Western diet induced an increase in the number and activation of liver recMφs in mice, conferring resistance to liver tumor metastasis due to increased IL-12 production by these macrophages. However, mice were susceptible to LPS-induced shock as a result of increased TNF-α production [11]. Further, recMφs and the TNF-α produced by them were deeply involved in the induction of NASH in fibroblast growth factor 5-null mice fed a Western diet, which was greatly improved by repeated low-dose irradiation [7]. We recently reported that liver LXRα KO mice fed a Western diet had increased numbers of liver recMφs with high TNF-α production, thereby developing NASH [12]. In contrast, concanavalin-A (Con-A) hepatitis is a mouse model of acute hepatic injury induced by NKT cells, recMφs, and resKCs, wherein ROS produced by resKCs are the final effectors [13]. Intriguingly, the vitamin D receptor (VDR), a member of the nuclear receptor superfamily, is highly expressed in resKCs, and VDR KO mice were resistant to Con-A hepatitis due to the resKCs in these mice exhibiting decreased ROS production even under enhanced recMφ TNF-α production [14]. Thus, VDR may differentially regulate the functions of resKCs and recMφs. These findings suggest that nuclear receptors, including LXRs, may be linked to metabolism and immune function in the liver through Kupffer cells/macrophages.

LXRα and LXRβ are members of the nuclear receptor superfamily of ligand-dependent transcription factors [15, 16]. LXRα is preferentially expressed in the liver, adipose tissue, and macrophages, whereas LXRβ is widely present throughout the body [15]. Both LXRs are activated by oxysterols, such as 24,25(*S*)-epoxycholesterol and 22(*R*)-hydroxycholesterol, and regulate the expression of genes involved in cholesterol and lipid metabolism, such as ATP-binding cassette A1, cholesterol 7α-hydroxylase, and sterol regulatory element-binding protein 1c (SREBP-1c) [16]. In addition, LXRs regulate immune cell responses, including those of macrophages and dendritic cells [17]. We previously reported that LXRβ is expressed in both resKCs and recMφs, while LXRα was predominantly expressed in resKCs [12]. In peritoneal macrophages, LXR activation suppressed LPS-induced acute inflammatory cytokine expression by inhibiting nuclear factor-κB transactivation [18]. Another study reported that LXR activation enhanced the phagocytosis of apoptotic cells by peritoneal Mφs while suppressing inflammatory gene expression [19].

Macrophages are thought to be involved in various aspects of lipid metabolism. One of their major functions in this context is the phagocytosis of excessive lipoproteins in serum. However, aberrant phagocytosis results in the internalization of excessive lipoproteins and the subsequent formation of foam cells, which exacerbate atherosclerosis and NASH [20, 21]. In these reports, CD36 is a primary scavenger receptor for oxidized low-density lipoprotein (LDL) and involves in this foam cell formation in NASH. Thus, endocytosis of denatured LDL and CD36 expression are the important functional criteria which evaluate the implication of macrophage in metabolic diseases.

In this study, we sought to elucidate how irradiation and LXR affect the functions of liver recMφs and resKCs, including bacteria phagolytic activity, cytokine production, and LDL endocytosis.

## Materials and methods

### Mice

C57BL/6J (WT) mice were obtained from SLC Japan (Hamamatsu, Japan). *Nr1h3*^-/-^ (*Lxrα*^-/-^, LXRα-KO) and *Nr1h3*^-/-^;*Nr1h2*^-/-^ (*Lxrα*^-/-^;*Lxrβ*^-/-^, LXRα/β-KO) mice were kindly provided by Dr. David J. Mangelsdorf (University of Texas Southwestern Medical Center) [22] and backcrossed with B6 mice for at least 10 generations. Mice were maintained under controlled temperature (23 ± 1°C) and humidity (45%–65%) with a 12-h light/dark cycle and free access to water and chow (CE-2; CLEA, Tokyo, Japan). Mice between 30 and 50 weeks of age were used to match the human age of over 40 years.

The Ethics Committee of Animal Care and Experimentation, National Defense Medical College, Japan, approved all requests for animals and the intended procedures for the present study (Permission number: 18064). All experiments were performed in accordance with the relevant guidelines and regulations. Every effort was made to minimize animal suffering, and there were no unexpected deaths.

### Reagents

T0901317 [N-(2,2,2-trifluoro-ethyl)-N-[4-(2,2,2-trifluoro-1-hydroxy-1-trifluoromethyl- ethyl)-phenyl]-benzenesulfonamide] was obtained from Cayman Chemical Company (Ann Arbor, MI, USA). *Escherichia coli*-derived LPS (O111: B4) was purchased from Sigma-Aldrich (St. Louis, MO, USA). Clodronate encapsulated in liposomes was obtained from FormuMax Scientific Inc. (Sunnyvale, CA, USA).

### Isolation of liver leukocytes

Mice were euthanized under deep anesthesia through inhalation of 5% isoflurane, and blood samples were obtained from the inferior vena cava into a heparinized syringe. Mice were perfused with 10 ml saline to remove blood from organs, and the liver was obtained. Liver non-parenchymal leukocytes were isolated via Percoll density gradient centrifugation, as previously described [8]. Briefly, the liver was minced with scissors and digested with 0.05% collagenase (Wako, Osaka, Japan). The digested liver was dispersed in medium by pushing it with syringe rubber on a stainless steel mesh (30 μm wire thickness and 72 μm pore size, AMICHU corporation, Osaka, Japan). Non-parenchymal MNCs were isolated from hepatocytes via Percoll density gradient centrifugation (33%), and red blood cells were removed using RBC lysis buffer.

### Flow cytometry

Liver leukocytes were incubated with Fc-blocker (BD Biosciences, Franklin Lakes, NJ, USA) to prevent non-specific binding and labeled with APC-CD45 (Thermo Fisher, Waltham, MA USA) and PerCP-eFluor710-Ly-6G (Thermo Fisher). To exclude non-immune cells and neutrophils, CD45^+^Ly6G^−^ mononuclear cells (MNCs) were gated. FITC-F4/80 and PE-CD11b (Thermo Fisher) were used to define each macrophage population. For the analysis of PE-Ly6c, PE-CD36 Ab (Thermo Fisher), and PE-MerTK (R&D Systems, Minneapolis, MN, USA), PerCP-Cy5.5-CD11b was selected to identify each cell population. The cells with the highest expression of CD11b in addition to high FS and SS were defined as neutrophils and were excluded from the analysis. In the case of CLEC4F (C-type lectin domain family 4 F) staining, cells with or without permeabilization of cell membranes were labeled with a polyclonal goat anti-mouse CLEC4F antibody (R&D Systems) and an FITC-donkey polyclonal anti-goat IgG antibody (R&D Systems). PE-F4/80 (Thermo Fisher) was used for macrophage detection. Similarly, after permeabilization of the cell membrane (BD Biosciences), intracellular TNF-α was labeled with FITC-anti-TNF (Thermo Fisher). The cells labeled with fluorescent antibodies were analyzed using a Novocyte flow cytometer (Agilent, Santa Clara, CA, USA).

### Bacteria phagocytic activity and acetylated LDL endocytosis

For the analysis of phagocytic activity, a total of 2.5×10^9^ FITC-*E*. *coli* (particles/kg, Thermo Fisher) were injected into mice, and liver MNCs were obtained 15 min after injection. The cells were labeled with PE-F4/80, PerCP-Cy5.5-CD11b, and APC-CD45 for the identification of resKCs and recMφs. The phagocytic activity of each cell population was evaluated based on the FITC signal intensity of non-injected control mice.

For the analysis of phagolytic activities, pHrodo^®^ E. coli (Thermo Fisher) was used *in vitro*. Its fluorescence emission increases as the pH of the surrounding environment becomes acidic, enabling the detection of functional phagolysosomes in phagocytic cells. Liver MNCs obtained from collagenase-treated livers (pretreated with 1 μM T0901317 or DEMSO for 18 h) were cultured with pHrodo^®^ *E*. *coli* (50 μL per 5 × 10^5^ cells) at 37 °C for 1 h, harvested by pipetting on ice, washed with medium, and then labeled with FITC-F4/80, PerCP-Cy5.5-CD11b, and APC-CD45. The phagolytic activity of each cell population was determined based relative to the PE signal intensity after being kept on ice for 1 h instead of at 37 °C.

To assess the *in vivo* endocytosis of modified-LDL, 100 μL of DIO (green fluorescence)-acetylated LDL (Thermo Fisher) or control acetylated LDL was injected intravenously into mice, and, 20 min after injection, the endocytosis by resKCs and recMφs was evaluated via flow cytometry.

### Viable *E*. *coli* challenge and plasma levels of TNF-α and IL-12

2.5 × 10^10^ CFU (/kg mouse) of K-12 strain *E*. *coli*. (ATCC, Manassas, VA, USA) were administered intravenously to mice, and blood samples were collected to measure the plasma cytokines (TNF-α ELISA kit; BD Biosciences, Total IL-12 ELISA Kit, Thermo Fisher).

### LXR ligand pretreatment of liver MNCs and their cytokine production after LPS stimulation

Hepatic MNCs were isolated from WT mice with collagenase treatment and incubated with T0901317 (1 μM) or control (DEMSO) for 18 h, followed by stimulation with LPS (1 ng/mL). After 45 min of LPS stimulation, mRNA was extracted from adherent cells using an RNeasy mini kit (Qiagen, Venlo, the Netherlands) and subjected to reverse transcription with SuperScript III (Invitrogen), followed by quantitative PCR analysis with LightCycler 480 SYBR Green I (Roche, Basel Switzerland). For the analysis of inflammatory cytokine production, liver MNCs isolated without collagenase treatment (lacking resKCs) were cultured with T0901317 or control (DEMSO) for 18 h and were subsequently stimulated with LPS. The supernatant of each well was collected after 6 h for TNF-α and 12 h for IL-12. The concentration of each cytokine was measured using ELISA (TNF-α ELISA kit; BD Biosciences, Total IL-12 ELISA Kit, Thermo Fisher).

### Radiation exposure

Radiation exposure was performed two days before sacrifice and immune cell isolation. The condition was set to 2 Gy, which is a relatively low dose, in order to prevent adverse effects on other organs as much as possible. The MBR-1503R (HITACHI, Tokyo, Japan) irradiation device was used at a dose rate of 0.4 Gy per minute. The physical condition of mice, including food intake, body weight, and moving activity, did not change after irradiation.

### Statistical analyses

Data are presented as the mean ± SE. We performed one-way analysis of variance (ANOVA) followed by Tukey’s multiple comparisons, unpaired Student’s *t*-tests, using the GraphPad software.

## Results

### Flow cytometric characterization of resKCs and recMφs

**Fig 1:**
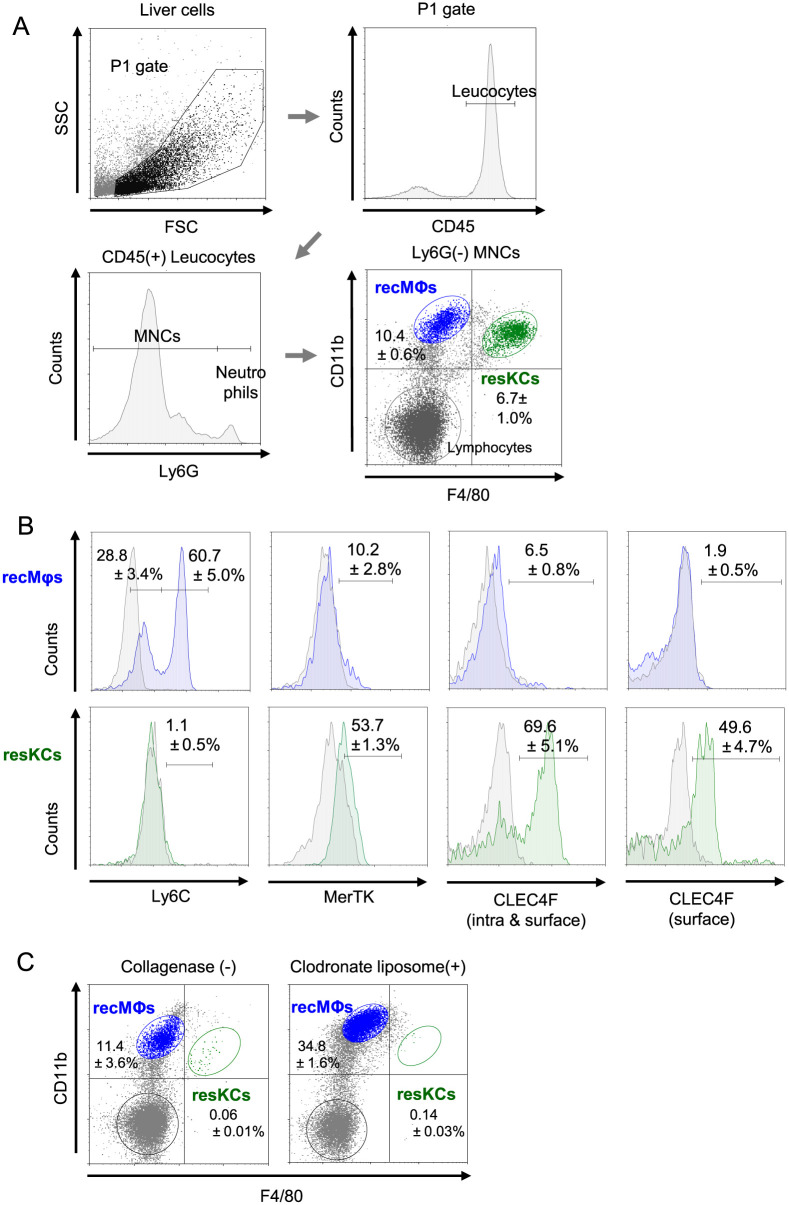
Flow cytometry characterization of resKCs and recMφs from mouse livers with or without collagenase pre-treatment. **(A)** Gating strategy of liver recMφs and resKCs with four-color analysis. Livers from B6 mice were minced and treated with collagenase. Non-parenchymal cells were extracted and subjected to flow cytometry analysis. The extracted cells were gated with FSC and SSC, and CD45-positive immune cells were selected. Ly6G-negative cells, excluding neutrophils, were used for the experiments. Based on FACS analysis of F4/80 and CD11b expression, F4/80^low^ CD11b^high^ cells were recMφs (blue dots)., and F4/80^high^ CD11b^low^ cells were defined as resKCs (green dots). (n = 13). **(B)** The expression of Ly6C, MerTK, as well as intracellular and surface CLEC4F in recMφs (upper panels with blue histogram) and resKCs (lower panels with green histogram) was demonstrated (n = 5). **(C)** The FACS analysis of liver MNCs obtained without collagenase treatment (left panel) and after clodronate liposome administration (200 μl per mouse) are displayed (right panel) (n = 6). Data are presented as the mean ± SE.

In WT mice, we defined recMφs as F4/80^low^ CD11b^high^ and resKCs as F4/80^high^ CD11b^low^ among CD45^+^ Ly6G^−^ liver mononuclear cells (MNCs) in the P1 gate (Fig 1 A) (n = 13). RecMφs are Ly6C^low or high^ (a marker of bone marrow-derived cells), while resKCs are Ly6C^−^ (Fig 1B). ResKCs are MerTK^+^ (a resident Mφ marker), while most recMφs do not express MerTK (Fig 1B), as we previously reported [2]. In addition, we assessed the expression of CLEC4F (C-type lectin receptor-4F, known as a Kupffer cell receptor), which is reportedly expressed in resKCs, but not in resident Mφs of other organs nor in bone marrow-derived Mφs [23, 24]. We found that resKCs were intracellular and surface CLEC4F-positive (n = 5) (Fig 1B) (n = 3), whereas liver recMφs were mostly negative for CLEC4F.

As we previously reported [3], resKCs were not obtained without collagenase treatment (Fig 1C) (n = 6). Clodronate liposome depleted resKCs and increased recMφs at 48 h after i.v. injection (Fig 1C) (n = 4), suggesting that phagocytic resKCs produce MCP-1 before undergoing apoptosis to recruit more recMφs into the liver [2].

We previously reported that CD68 is a marker of resKCs, with recMφs having intracellular CD68 and activated recMφs expressing surface CD68 [3, 7]. In contrast to CD68, activation of recMφs via LPS injection into mice did not induce CLEC4F according to the previous report [23]. These results indicate that F4/80 and CD11b in combination with Ly6c and CLEC4F may be a robust markers for the classification of resKCs and recMφs.

### Differences in radiosensitivity between resKCs and recMφs

**Fig 2:**
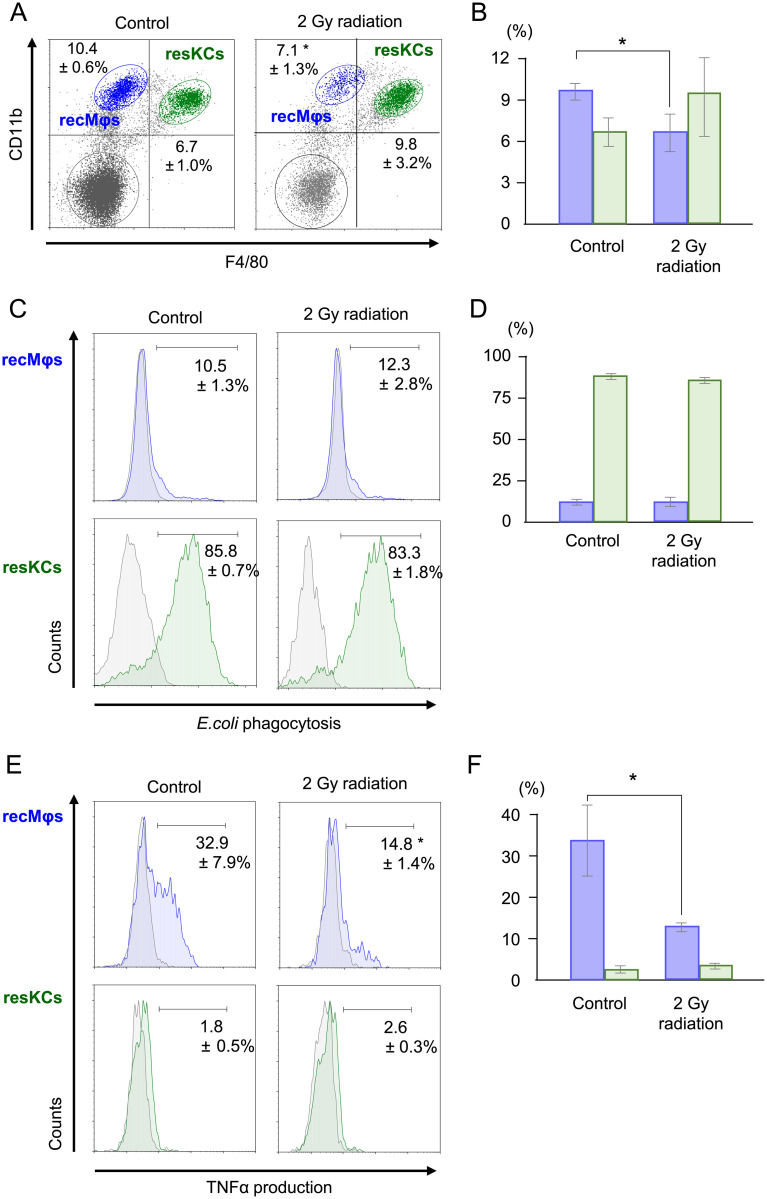
Effect of low-dose irradiation on the proportion and function of resKCs and recMφs in WT mice. Irradiation-induced changes in the proportion and functions of resKCs and recMφs. Liver MNCs harvested from normal mice and mice 2 days after irradiation (2 Gy) were analyzed. **(A)** FACS analysis of F4/80 and CD11b in liver MNCs isolated from control and irradiated mice, with recMφs and resKCs displayed as blue and green dots, respectively. **(B)** Bar graph of recMφ (blue columns) and resKC (green columns) abundance in control and irradiated mice (n = 13 and 6). **(C)** Phagocytic activity of recMφs (upper panels) and resKCs (lower panels) against intravenously injected FITC-*E*. *coli* in mice with or without irradiation. The percentage of FITC-*E*. *coli*-positive cells was calculated relative to non-injected control mice (gray area in each panel). **(D)** Bar graph of *E*. *coli* phagocytosis by recMφs (blue columns) and resKCs (green columns) in control and irradiated mice (n = 5 in each group). **(E)** Intracellular TNF-α production in recMφs (upper panels) and resKCs (lower panels) stimulated with LPS *in vitro*. Liver MNCs from control or irradiated mice were stimulated with LPS *in vitro* for 1.5 h, and intracellular TNF-α was labeled (n = 5 in each group). **(F)** Bar graph of intracellular TNF-α expression in recMφs (blue columns) and resKCs (green columns) in control and irradiated mice (n = 5 in each group). Data are presented as the mean ± SE (*p <0.05, unpaired Student’s *t*-test).

To further clarify the functional differences between resKCs and recMφs [7], low-dose irradiation (2 Gy) was performed in WT mice. The distribution and functions of resKCs and recMφs were examined 2 days after irradiation. Irradiation decreased the total number of liver MNCs compared to control mice without irradiation (6.2±0.4×10^6^ vs. 2.3±0.3×10^6^ cells, n = 13, 6) (P<0.01). It also proportionally decreased recMφs (10.4% vs. 7.1%) (p<0.05) (Fig 2A and 2B) among liver MNCs. ResKCs, but not recMφs, showed potent *in vivo* phagocytosis of FITC-*E*. *coli* (15 min after i.v. injection), and irradiation did not significantly decrease their phagocytic activity (Fig 2C and 2D, n = 5). In contrast, when liver MNCs from mice with or without irradiation were stimulated with LPS *in vitro*, a certain proportion of recMφs (32.9±7.9%), but not resKCs, produced intracellular TNF-α, while irradiation significantly decreased the proportion of intracellular TNF-α-positive recMφs (14.8±1.4%) (Fig 2E and 2F, n = 5, p<0.05). These results suggest that irradiation impaired the inflammatory function of recMφs but did not substantially affect the phagocytic activity of resKCs.

### Synthetic LXR ligand (T0901317) pretreatment *in vitro* enhanced the phagocytic activity of resKCs but decreased LPS-induced TNF-α production by recMφs

**Fig 3:**
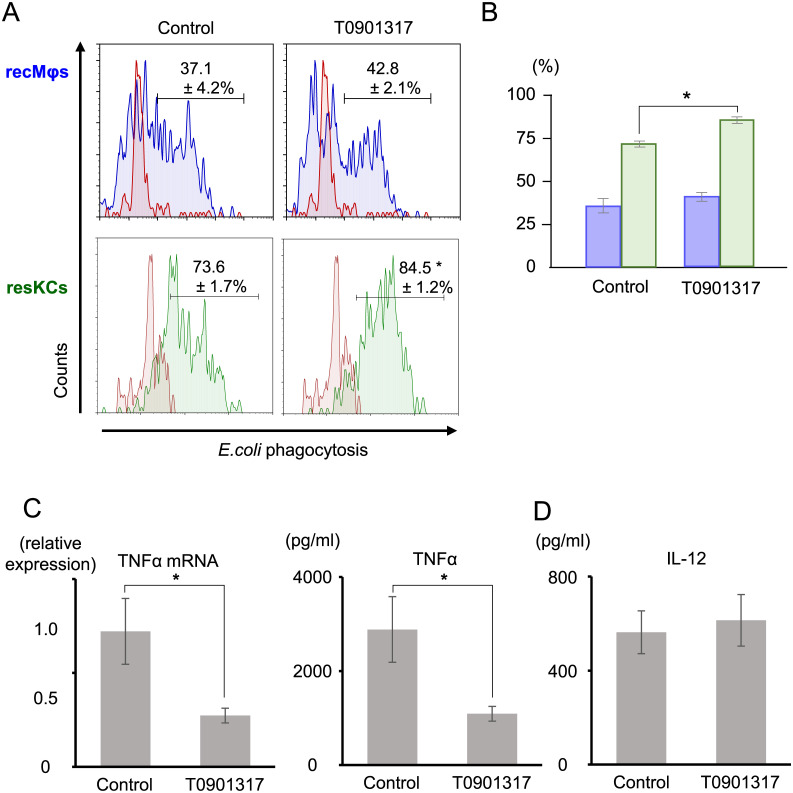
The increased *in vitro* phagocytic activity of resKCs and recMφs against FITC-*E*. *coli* and the decreased TNF-α production by LPS-stimulated liver MNCs following pre-treatment with a synthetic LXR ligand (T0901317). **(A)** The phagocytic activity of recMφs (upper panels, blue line) and resKCs (lower panels, green line) against *E*. *coli*. Negative controls were incubated with *E*. *coli* for 1 h on ice (red line). **(B)** Bar graph of the *in vitro* phagocytic activities of recMφs (blue columns) and resKCs (green columns) in control and T0901317-treated MNCs (n = 12 in each group). **(C)** The *in vitro* TNF-α production by MNCs with or without T0901317 pre-treatment. Adherent cells were obtained after incubation with LPS for 45 minutes, mRNA was isolated, and the relative expression TNF mRNA was compared between the two groups (left panel, n = 5 in each group). After incubation with LPS for 6 h, culture supernatant was collected, and TNF-α concentration was measured and compared (left panel, n = 14 in each group). **(D)** IL-12 production by liver MNCs stimulated with LPS for 12 h with or without T0901317 pre-treatment (n = 9 in each group). Data are presented as the mean ± SE (*p <0.05, unpaired Student’s *t*-test).

Liver MNCs were isolated after collagenase treatment and pretreated *in vitro* with T0901317 or control (DEMSO) overnight (18 h). Thereafter, liver MNCs were cultured with FITC-*E*. *coli* for 1 h. MNCs were harvested from the plates and analyzed via flow cytometry. The results showed that liver resKCs pretreated with T0901317 had more potent phagocytic activity against *E*. *coli* than the control resKCs (Fig 3A and 3B, green lines and columns, n = 7). RecMφs exhibited weaker phagocytic activity against FITC-*E*. *coli* than resKCs. After T0901317 pretreatment, the difference in their phagocytic activity was not statistically significant (Fig 3A and 3B, blue lines and columns, n = 7). In contrast, the mRNA expression of TNF in T0901317-pretreated liver MNCs following LPS stimulation *in vitro* was significantly lower compared to that of control liver MNCs (Fig 3C left panel, n = 5 in each). Consistently, hepatic MNCs pretreated with T0901317 produced less TNF-α after LPS stimulation than control hepatic MNCs (Fig 3C, right panel, n = 14 in each). Since hepatic MNCs harvested without collagenase treatment included virtually no resKCs, TNF-α was mainly produced by LPS-stimulated recMφs. However, T0901317 pretreatment did not affect IL-12 production by recMφs (Fig 3D, n = 9 in each group). These results suggest that LXR signaling enhanced the phagocytic function of resKCs but inhibited the inflammatory activity of recMφs.

### LXRα/β KO mice showed a decreased population of resKCs with impaired phagocytic activity in parallel to an increased recMφs population with enhanced TNF-α production

**Fig 4:**
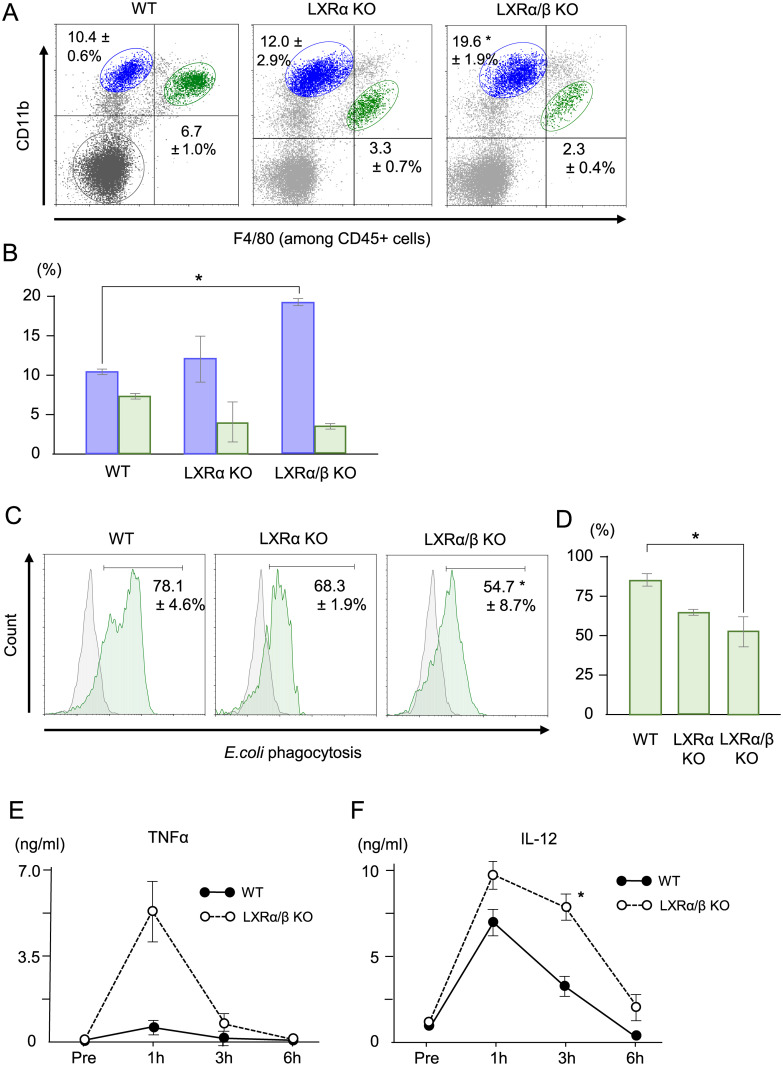
The proportions of resKCs and recMφs, *in vivo* phagocytic activity of resKCs against *E*. *coli*, and plasma inflammatory cytokine levels in WT and LXR-deficient mice. **(A)** The proportions of resKCs and recMφs in the livers of WT, LXRα KO, and LXRα/β KO mice were determined via flow cytometry (n = 4 in each group). **(B)** Bar graph of recMφ (blue columns) and resKC (green columns) proportions in WT, LXRα KO, and LXRα/β KO mice (n = 4 in each group) Data are presented as the mean ± SE (*p <0.05, one-way ANOVA followed by Tukey’s multiple comparisons). **(C)** *In vivo* phagocytic activity of WT mice (n = 6), LXRα KO mice (n = 3), and LXRα/β KO mice (n = 3). Mice were injected with FITC-*E*. *coli*, and the phagocytic activity of resKCs (green line) was analyzed 15 min after injection. ResKCs from a mouse without *E*. *coli* injection were used as negative controls (gray line). **(D)** Bar graph of the phagocytotic activity of resKCs (green columns) in WT, LXRα KO, and LXRα/β KO mice (n = 6, 3, 3 in each). Data are presented as the mean ± SE (*p <0.05, one-way ANOVA followed by Tukey’s multiple comparisons) **(E, F)** WT mice and LXRα/β KO mice were injected with live *E*. *coli*, and plasma TNF-α and IL-12 levels were measured at the indicated time points after *E*. *coli* injection (n = 3 in each group). Data are presented as the mean ± SE. (*p <0.05, unpaired Student’s *t*-test).

LXRα/β KO mice had higher proportions of recMφs and lower proportions of resKCs than WT mice (p<0.05) (Fig 4A and 4B, n = 4). ResKCs of LXRα/β KO mice exhibited significantly lower phagocytic activity against FITC-*E*. *coli* at 15 min after injection compared to those of WT mice, while the phagocytic activity of LXRα KO mouse resKCs did not differ significantly compared to that of WT mouse resKCs (Fig 4C and 4D, n = 3 each). LXRα/β KO mice had much higher plasma TNF-α levels at 1 h after live *E*. *coli* injection than WT mice (Fig 4E) (n = 3 in each group), suggesting that recMφs in the liver and other organs were activated and had a high TNF-α production capacity. Plasma IL-12 levels in LXRα/β KO mice at 12 h after viable *E*. *coli* injection were also significantly higher than those in WT mice, although the difference was not as pronounced as observed for TNF-α levels (Fig 4F).

### Decreased phagolytic activity of resKCs against *E*. *coli* in LXRα KO mice

**Fig 5:**
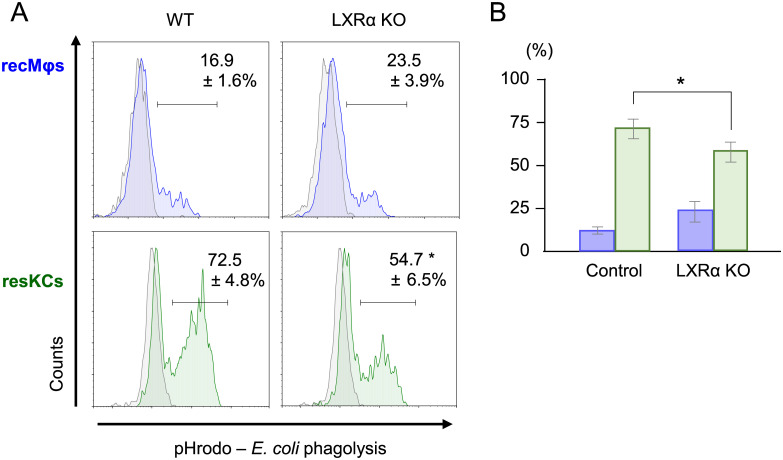
The decreased *in vitro* phagolytic activity of resKCs from LXRα KO mice against pHrodo *E*. *coli*. **(A)** The phagolytic activity of recMφs (upper panels, blue line) and resKCs (lower panels, green line). WT and LXRα KO mouse recMφs and resKCs incubated with pHrodo *E*. *coli* on ice were used as negative controls (gray line). **(B)** Bar graph of the phagolytic activity of recMφs (blue columns, n = 4) and resKCs (green columns, n = 4). Data are presented as the mean ± SE. (*p <0.05, unpaired Student’s *t*-test).

To further explore the role of LXRα in macrophage phagocytic function against *E*. *coli*, we assessed functional phagolysosome formation in resKCs using pHrodo *E*. *coli*. Interestingly, the *in vitro* positivity for pHrodo *E*. *coli* in resKCs from LXRα KO mice was significantly lower compared to that in WT mice (Fig 5A and 5B, n = 5 in each group), suggesting that LXRα is required for the phagolytic activity of phagolysosomes in resKCs. In addition, only a small proportion of recMφs were positive for pHrodo *E*. *coli*, suggesting that even though a certain proportion of recMφs phagocytosed *E*. *coli* (Fig 3A), they may not kill *E*. *coli* as effectively as resKCs.

### Decreased modified-LDL endocytosis by resKCs in LXRα KO and LXRα/β KO mice

**Fig 6:**
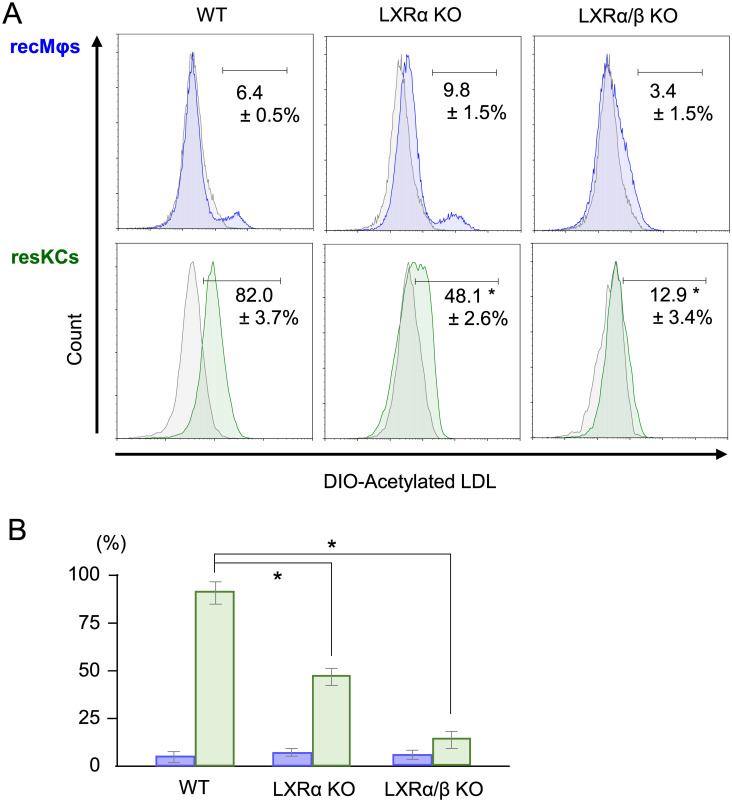
The decreased *in vivo* phagocytic activity of resKCs for acetylated LDL in LXRα KO and LXRα/β KO mice. **(A)** WT mice (n = 6), LXRα KO mice (n = 3), and LXRα/β KO mice (n = 3) were injected with DIO-labeled acetylated LDL, and the endocytosis of acetylated LDL by recMφs (upper panels, blue line) and resKCs (lower panels, green line) was analyzed via flow cytometry. ResKCs and recMφs from WT mice injected with control acetylated LDL (without fluorescence) were used as negative controls (gray line). **(B)** Bar graph of DIO-labeled acetylated LDL endocytosis by recMφs (blue columns) and resKCs (green columns). Data are presented as the mean ± SE (*p <0.05, one-way ANOVA followed by Tukey’s multiple comparisons test).

At 20 min after the intravenous injection, endocytosis activity of resKCs for acetylated LDL was blunted in LXRα KO mice and severely impaired in LXRα/β KO mice, when compared to WT mice (Fig 6A lower panels and Fig 6B green columns). These results suggested that LXR regulates not only the phagocytosis of bacteria, but also that of lipids. In contrast, recMφs in each group of mice endocytosed a small amount of acetylated LDL (Fig 6A upper panels and Fig 6B blue columns, n = 5). These results suggested that LXRα deletion may impair LDL endocytosis (as well as bacteria phagolysis capacity) in resKCs and subsequently reduce the hydrolysis of cholesterol esters and triglycerides in the phagolysosomes of resKCs [25], suggesting that lipogenesis was impaired in LXR KO mice.

### Increased CD36^+^ resKCs in LXRα KO and LXRα/β KO mice

**Fig 7:**
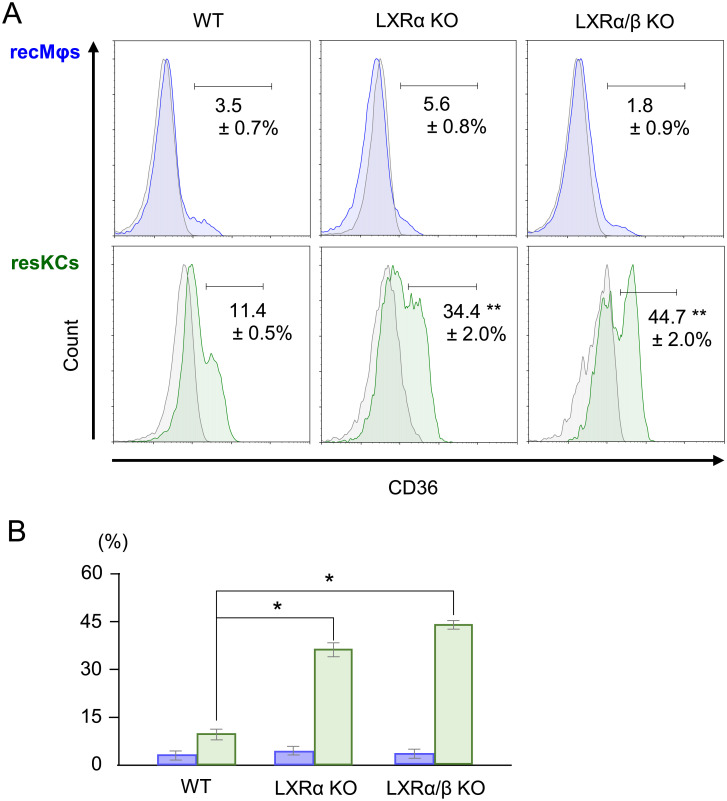
CD36 expression in resKCs and recMφs from WT, LXRα, and LXRα/β KO mice. **(A)** The expression of CD36 on recMφs and resKCs from WT, LXRα, and LXRα/β KO mice (n = 4 in each group) was analyzed and compared. **(B)** Bar graph of CD36^+^ cells among recMφs (blue columns) and resKCs (green columns). Data are presented as the mean ± SE. (*p <0.05, one-way ANOVA followed by Tukey’s multiple comparisons test).

The proportion of CD36^+^ resKCs in WT mice was higher than that of recMφs (Fig 7A, n = 6). Unexpectedly, the number of CD36^+^ resKCs in LXRα KO and LXRα/β KO mice (n = 4, each group) was significantly higher than that in WT mice (Fig 7A) (p<0.01). Since LXR is a nuclear receptor implicated in the expression of CD36 (oxidized LDL receptor) [26], it was somewhat unexpected that the number of CD36^+^ resKCs in LXRα and LXRα/β KO mice was higher than that in WT mice. However, peroxisome proliferator-activated receptor γ (PPAR-γ) is considered the main inducer of CD36 [27]. Therefore, CD36 expression may be upregulated to compensate for the impaired LDL uptake by resKCs in LXR KO mice.

## Discussion

The present study provides insight into the function of resKCs and recMφs, with LXRs affecting these two subsets in contrasting manners. LXR stimulation potentiated the phagocytic function of resKCs but suppressed TNF-α production by recMφs. LXRs may also be involved in lipid metabolism through regulating LDL endocytosis by resKCs. Taken together, LXRs modulated the function of both subsets in a sophisticated manner to maintain host defense against infections and metabolic dysregulation.

Since the identification of resKCs and recMφs [3], we have realized that the immunological phenomena in the liver cannot be elucidated without distinguishing the roles of these two subsets. ResKCs are essential for anti-bacterial immunity through their phagocytic activity, whereas recMφs are essential for anti-tumor immunity through IL-12 production [2, 3, 7]. Consistently, the depletion of resKCs by clodronate liposome treatment rendered mice extremely susceptible to bacterial infections, whereas liver anti-tumor immunity was not compromised at all (rather, it was enhanced) as the number of recMφs was increased, presumably due to resKCs producing MCP-1 prior to apoptosis [2]. Non-lethal irradiation was previously reported to transiently decrease recMφs, but not resKCs [2]. In the current study, 2 Gy irradiation diminished TNF-α production in LPS-stimulated recMφs but did not affect resKC phagocytic activity against *E*. *coli*. Consistently, the depletion of recMφs by repeated low-dose irradiation (1 Gy/week for 8 weeks) greatly decreased plasma TNF-α levels and remarkably improved NASH in fibroblast growth factor-5-null mice fed a Western diet [7]. In previous reports, radiation could induce TNF-α secretion, which was in turn implicated in radiation-induced fatal organ inflammation [28]. In these experiments, the amount of radiation was over 10 Gy, which can cause damage to various cellular components of tissue. In contrast, low-dose radiation affects cells with shorter turnover rates, such as bone marrow-derived immune cells, and reportedly inhibits inflammatory reactions [29], including TNF-α secretion by macrophages. RecMφs are implicated in experimental models of acute hepatic injury induced by α-galactosylceramide (α-GalCer) and CpG-ODN [2]. FasL-producing NKT cells stimulated by recMφ-secreted TNF-α function as the final effectors in α-GalCer hepatitis and CpG-ODN hepatitis, especially in mice fed a Western diet [2]. However, in the case of acute Concanavalin-A hepatitis, activated resKCs act as the final effectors through TNF-α-induced ROS production [13, 14].

LXR ligand pre-treatment was reported to enhance host immunity against bacterial infections [30]. In addition, LXRα/β KO mice, as well as LXRα KO mice, but not LXRβ KO mice, were reported as susceptible to bacterial infections [30]. In line with these observations, LXRα KO but not LXRβ KO mice fed a Western diet were susceptible to hepatic cholesterol overload and NASH [22, 31]. These findings suggest that LXRα, rather than LXRβ, is preferentially involved in the phagocytosis of bacteria and lipid endocytosis. Furthermore, LXR ligands reduced TNF-α production by Kupffer cells (presumably recMφs) in mice injected with LPS, attenuating LPS-induced liver injury [32]. In line with these observations, we recently reported that the number of liver recMφs was increased in LXRα/β KO mice, and LPS-induced liver injury was exacerbated in mice due to the increased numbers of recMφs and associated TNF-α production [12]. Of note, the immunological alterations in LXR-deficient mice may be attributed to the indirect effects of LXR deficiency, such as compensatory reactions. Further studies in which synthetic ligands are administered to LXR-knockout mice are therefore needed to address this issue.

We recently reported that LXRα KO mice fed a Western diet had significantly increased numbers of liver TNF-α-producing recMφs and developed NASH, showing higher liver and plasma cholesterol levels but not liver triglyceride levels compared to WT mice fed a Western diet, in addition to lower plasma triglyceride levels [12]. Taken together with the current results, it is possible that LXRα signals are involved in cholesterol metabolism and lipogenesis in resKCs and suppress inflammation, including NASH-associated inflammation, by inhibiting TNF-α-producing recMφs. Therefore, LXRα deletion may impair LDL endocytosis in resKCs and subsequently reduce the hydrolysis of cholesterol esters and triglycerides in the phagolysosomes of resKCs [25, 33], suggesting that lipogenesis is impaired in LXRα KO mice. However, since cholesterol efflux and elimination are impaired in the resKCs and hepatocytes of LXRα KO mice fed a Western diet due to the ABC transporter deficiency [12], cholesterol may be stored in resKCs as well as hepatocytes. Thus, LXRα dysfunction could underpin the decrease in lipogenesis and increase of cholesterol accumulation, especially within the liver (hepatocytes and resKCs).

It should be noted that the number and function of resKCs and recMφs may be influenced by various background factors, such as age, dietary status, chronic inflammation, and sex. These should be considered during research. In humans as well as mouse experimental models, metabolic syndrome and tissue damage usually occur in elderly individuals (≥20 weeks old in mice and ≥40 years old in humans) and progress with aging [34–36]. Therefore, we used aged mice (30–50 weeks of age) in the current study.

The LXR-enhanced phagolytic activity of resKCs and decreased TNF-α production by recMφs are both beneficial for host protection against bacterial infections and TNF-α-mediated metabolic disease (diabetes, atherosclerosis, NASH, etc.). Notably, the administration of LXR ligands also activates lipogenesis by enhancing hepatic fatty acid synthesis through the upregulation of sterol regulatory element-binding protein 1c (SREBP-1c) and carbohydrate response element-binding protein (ChREBP), resulting in hypertriglyceridemia and hepatic steatosis [37–39]. These adverse metabolic effects impede the application of LXR ligands in clinical practice. A previous report revealed that LXR ligand treatment could ameliorate inflammatory reactions while preventing hypertriglyceridemia [40]. An approach for upregulating endogenous LXR ligand desmosterol was recently published [41]. It could suppress inflammatory reactions while ameliorating fat synthesis via SREBP inhibition. Based on these reports, LXR stimulation is a promising strategy for the treatment of various inflammatory diseases. Therefore, the elucidation of its effect on immune cells and metabolism, with a focus on different macrophage populations, is essential for future immuno-metabolism research.
